# Therapeutically engineered induced neural stem cells are tumour-homing and inhibit progression of glioblastoma

**DOI:** 10.1038/ncomms10593

**Published:** 2016-02-02

**Authors:** Juli R. Bagó, Adolfo Alfonso-Pecchio, Onyi Okolie, Raluca Dumitru, Amanda Rinkenbaugh, Albert S. Baldwin, C. Ryan Miller, Scott T. Magness, Shawn D. Hingtgen

**Affiliations:** 1 Division of Molecular Pharmaceutics, UNC Eshelman School of Pharmacy, The University of North Carolina at Chapel Hill, Chapel Hill, North Carolina 27599, USA; 2 Department of Genetics, UNC Human Pluripotent Stem Cell Core, UNC School of Medicine, The University of North Carolina at Chapel Hill, Chapel Hill, North Carolina 27599, USA; 3 Neuroscience Center, School of Medicine, The University of North Carolina at Chapel Hill, Chapel Hill, North Carolina 27599, USA; 4 Lineberger Comprehensive Cancer Center, The University of North Carolina at Chapel Hill, Chapel Hill, North Carolina 27599, USA; 5 Division of Neuropathology, Department of Pathology and Laboratory Medicine, UNC School of Medicine, The University of North Carolina at Chapel Hill, Chapel Hill, North Carolina 27599, USA; 6 Department of Neurology, UNC School of Medicine, The University of North Carolina at Chapel Hill, Chapel Hill, North Carolina 27599, USA; 7 Department of Cell Biology and Physiology, UNC School of Medicine, The University of North Carolina at Chapel Hill, Chapel Hill, North Carolina 27599, USA; 8 Biomedical Research Imaging Center, The University of North Carolina at Chapel Hill, Chapel Hill, North Carolina 27599, USA

## Abstract

Transdifferentiation (TD) is a recent advancement in somatic cell reprogramming. The direct conversion of TD eliminates the pluripotent intermediate state to create cells that are ideal for personalized cell therapy. Here we provide evidence that TD-derived induced neural stem cells (iNSCs) are an efficacious therapeutic strategy for brain cancer. We find that iNSCs genetically engineered with optical reporters and tumouricidal gene products retain the capacity to differentiate and induced apoptosis in co-cultured human glioblastoma cells. Time-lapse imaging shows that iNSCs are tumouritropic, homing rapidly to co-cultured glioblastoma cells and migrating extensively to distant tumour foci in the murine brain. Multimodality imaging reveals that iNSC delivery of the anticancer molecule TRAIL decreases the growth of established solid and diffuse patient-derived orthotopic glioblastoma xenografts 230- and 20-fold, respectively, while significantly prolonging the median mouse survival. These findings establish a strategy for creating autologous cell-based therapies to treat patients with aggressive forms of brain cancer.

## Abstract (web summary)

Neural stem cells have a tropism for glioblastoma. Here the authors employ fibroblasts directly reprogrammed into induced neural stem cells and loaded with cytotoxic molecules to migrate to xenotransplanted brain tumours in mice, achieving tumour shrinkage and prolonged survival.

## Graphical abstract

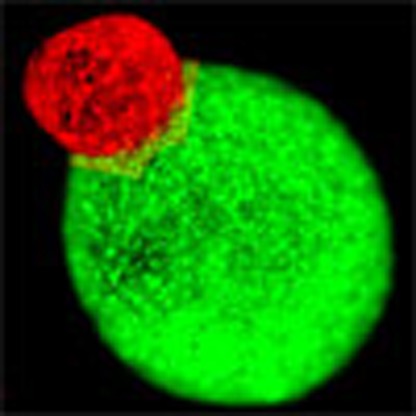

Ever since Yamanaka published his groundbreaking studies on induced pluripotent stem cells (iPSCs)^1^,^2^, cellular reprogramming has opened new avenues for potential transplantation therapies^3^. However, reports that iPSCs form cancerous teratomas when implanted *in vivo* have limited the application of iPSC and iPSC-derived cell transplant therapies. With the recent development of transdifferentiation (TD), where somatic cells are directly reprogrammed into an alternate lineage bypassing dedifferentiation into a pluripotent state, reprogramming technology now stands poised to achieve safe personalized cell transplant therapy^4^. The newest addition to the cell types created by TD is neural stem cells (NSCs), called induced NSCs (iNSCs)^5^,^6^,^7^,^8^. iNSCs were first reported by Kim *et al.*^5^, followed closely by several studies using different TD strategies^6^,^7^,^9^. In these studies, iNSCs were found to express nestin and differentiate into astrocytes, neurons and oligodendrocytes similar to brain-derived NSCs. Yet, unlike NSCs derived from embryonic stem cells or iPSCs, iNSCs showed no cancerous teratoma formation *in vivo*^7^,^10^. This suggests that iNSCs can provide safe, routine, patient-specific cell transplantation therapy to treat disorders of the central nervous system (CNS). However, the efficacy of iNSC-based therapy remains to be defined.

Cancers of the CNS are devastating diseases. Each year, 14,000 patients are diagnosed with glioblastoma (GBM), the most common primary brain cancer^11^. Despite multimodal treatment, the median life expectancy for GBM patients is only 15–17 months, and fewer than 10% will survive beyond 5 years (ref. 12). NSC delivery of therapeutic molecules is emerging as a promising approach to achieve effective killing of cancers where conventional treatments fail^13^,^14^,^15^,^16^,^17^,^18^. NSCs display unique tumouritropic migration that allows them unprecedented access to solid and invasive cancer cells^18^. In addition, when stably engineered to release anticancer molecules, NSCs provide long-term drug delivery directly to cancerous deposits. This exceptional tropism towards areas of pathology and highly selective drug delivery directly into tumours has allowed tumouricidal NSC therapies to exhibit potent anticancer efficacy in a variety of preclinical models^13^,^14^,^15^,^16^. Despite these successes, procurement of appropriate cells for routine clinical use remains one of the greatest challenges to tumouricidal NSC therapy. The ideal cell carrier would be autologous and readily available; however, NSCs reside deep within the adult brain in limited quantities^19^. The properties of iNSCs make them an attractive source to derive personalized cellular vehicles for cancer therapy. In addition to their unique ability for safe patient-specific generation, iNSCs display the key attributes of effective cell carrier systems: high expression of engineered transgenes, long-term survival *in vivo* and absence of tumour formation^4^,^5^,^7^,^8^. This suggests that a new class of autologous NSC-based cancer therapies could be created by transdifferentiating skin fibroblasts into iNSCs that could be expanded, engineered and re-implanted into patients.

In this study, we provide the first insights into iNSC-based therapy by developing and testing the first iNSC-based drug-delivery vehicles. Using a combination of molecular assays, non-invasive serial imaging and human GBM xenografts, we were able to define (i) the survival and fate of these cells *in vivo*, (ii) the tumouritropic migration of this novel drug carrier and (iii) the efficacy of iNSC-based tumouricidal therapy against solid and diffuse brain cancer in preclinical models.

## Results

### Engineered iNSCs differentiate and proliferate

**Figure 1:**
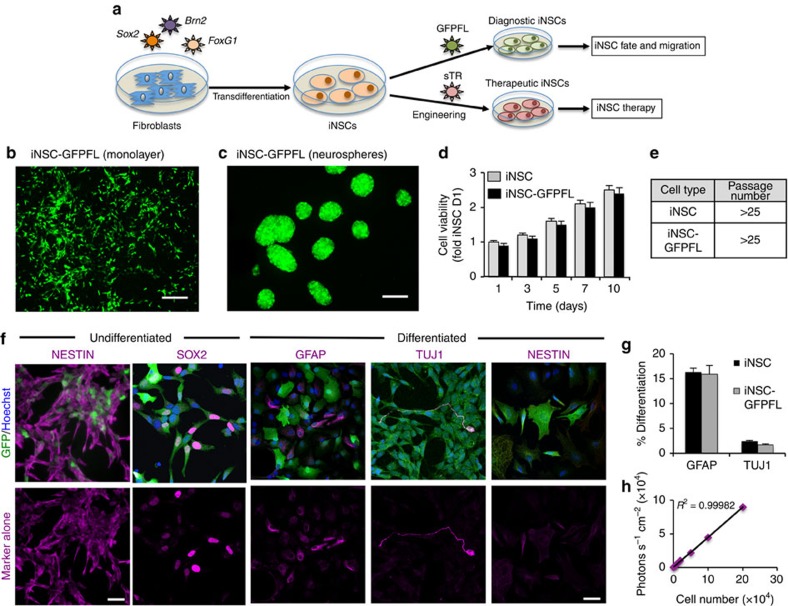
Generation and characterization of diagnostic and therapeutic iNSCs. (**a**) Schematic depiction of the strategy used to create therapeutic and diagnostic variants of iNSCs. (**b**,**c**) Representative fluorescent photomicrographs of iNSCs engineered to express GFPFL and grown as monolayers (**b**) or neurospheres (**c**). (**d**) Summary graph showing the growth of GFPFL-expressing iNSCs in comparison with unmodified iNSCs. (**e**) Summary table showing the maximum passage number of iNSCs expressing GFPFL and unmodified iNSCs. (**f**) Representative images of immunofluorescence that show the expression of the NSC markers nestin and Sox2 (staining shown in magenta) in iNSC-GFPFL (green). In addition, iNSC-GFPFLs were differentiated by mitogen removal and culturing for 12 days. The cells were stained to detect GFAP+ astrocytes, Tuj-1+ neurons and nestin (staining shown in magenta). Fluorescent images showing only the red (555 nm) secondary antibody channel are shown in the bottom row. (**g**) Quantification of GFAP+ or Tuj-1+ cells present after differentiation of iNSC-GFPFL or unmodified iNSCs. (**h**) Summary data showing the linear correlation between iNSC-GFPFL cell number and bioluminescence signal. iNSC-GFPFLs were plated at increasing cell numbers, combined with D-luciferin and were imaged in a luminometer (*R*^2^=0.99982). Scale bars in **b**,**c**, 100 and 20 μm in **f**. Data are mean±s.e.m. Data in **d**,**e**,**g**,**h** are from three to four independent experiments. In **d**, *P*>0.05 by two-way ANOVA.

To first generate iNSCs, we followed the method of Lujan *et al.* and transduced fibroblasts with lentiviral vectors (LV) encoding the transcription factors *Brn2*, *Sox2* and *FoxG1* (ref. 6; Fig. 1a). To permit the exploration of iNSC carrier fate, migration and anticancer efficacy, we next created a panel of iNSC cell carriers by using LVs to genetically engineer cells with optical reporters and therapeutic transgenes (Fig. 1a). We first evaluated whether general stem cell properties would be affected by LV modification. iNSCs cultured in a monolayer were transduced with LV encoding a green fluorescent protein (GFP)-luciferase fusion protein (iNSC-GFPFL). Forty-eight hours post transduction, robust GFP expression was observed in the cells (Fig. 1b). When iNSC-GFPFLs were placed in non-adherent flasks, the cells rapidly formed GFP+ neurospheres (Fig. 1c). Comparison of cell viability revealed minimal differences in the growth rate between iNSC-GFPFL and unmodified iNSCs through 10 days (2.6- versus 2.5-fold on day 10; Fig. 1d), and both cell lines were able to be extensively passaged in culture (Fig. 1e). As shown in Fig. 1f, iNSC-GFPFL robustly expressed the NSC markers nestin and Sox2. When induced to differentiate, iNSC-GFPFL formed GFAP+ astrocytes and Tuj-1+ neurons while simultaneously decreasing the expression of nestin. The generation of astrocytes and neurons by iNSC-GFPFL was as efficient as unmodified iNSCs (Fig. 1g and Supplementary Fig. 1). We next performed cytogenetic analysis on G-banded metaphase spreads from the iNSC-GFPFL. The analysis showed that the modified iNSCs exhibited a normal female karyotype (Supplementary Fig. 2). Lastly, a linear correlation between iNSC-GFPFL cell number and luciferase signal was observed *in vitro* (Fig. 1h).

**Figure 2:**
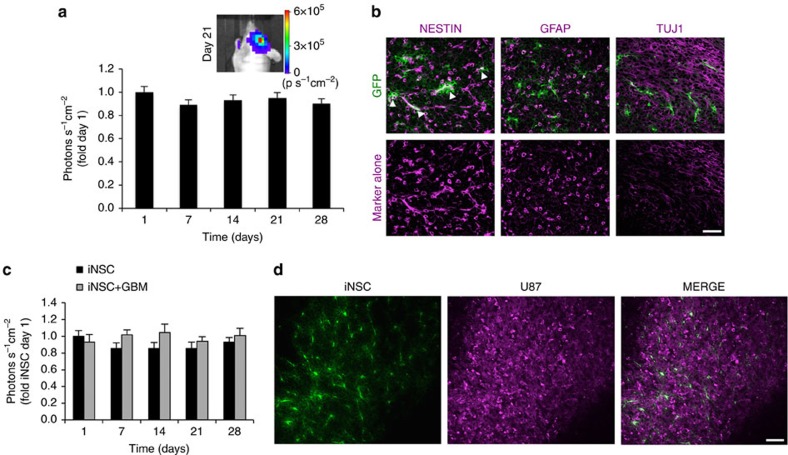
***In vivo*****characterization of iNSCs transplanted in the mouse brain in the presence or absence of GBM.** iNSC-GFPFLs were implanted into the frontal lobe of mice in the presence or absence of U87 human GBM (*n*=6). Serial bioluminescence imaging was used to monitor the volumes of iNSC-GFPFL. Two weeks after implantation, a subset of mice was killed and their brains sectioned and analysed. (**a**) Summary graph depicting the volume of iNSC-GFPFL in the brain in the absence of GBM through 28 days. (**b**) Immunofluorescence analysis of iNSC-GFPFL (green) 14 days post implantation into the brain. Nestin+ iNSC-GFPFLs were detected (indicated by arrowheads), and iNSC-GFPFL differentiation was detected with GFAP or Tuj-1 staining shown in magenta. Representative fluorescent images showing only the red (555 nm) secondary antibody channel are shown in the bottom row. (**c**) Engraftment of iNSC-GFPFL cells implanted in the brain in the presence or absence of human GBM (*n*=10 per group) measured using bioluminescence imaging. (**d**) Representative fluorescence imaging of post-mortem tissue sections showing GFP+ iNSCs (green) are still present in mCherry+ GBMs (magenta) 28 days after implantation. Scale bars in **b**,**d**, 40 and 50 μm, respectively. Data are mean±s.e.m. In **a**, *P*>0.05 by repeated measures one-way ANOVA. In **c**, *P*>0.05 by repeated measures two-way ANOVA.

We next utilized the engineered iNSCs to investigate the fate of these cells in the context of GBM. iNSCs have been reported to persist in the brain through 10 weeks post implantation^6^. We implanted iNSC-GFPFL into the parenchyma of mice in the presence and absence of GBMs. Using real-time non-invasive imaging to monitor the survival of engineered iNSCs, we found that iNSC levels did not statistically change through 4 weeks (Fig. 2a). Post-mortem immunohistochemistry (IHC) revealed that the majority of iNSC-GFPFL expressed the NSC marker nestin 2 weeks post implantation (Fig. 2b). We observed few iNSC-GFPFL cells expressing the astrocyte marker glial fibrillary acidic protein (GFAP) or the neuronal marker Tuj-1. Serial imaging also revealed that iNSC survival was not altered by implantation into the brains of mice bearing GBM (Fig. 2c). Using fluorescence imaging of tissue sections, we found that GFP+ iNSCs were still present in the mCherry+ GBM 28 days after implantation (Fig. 2d). Together, these results suggest that iNSCs can be genetically modified with minimal impact on their capacity to proliferate or differentiate, and that the modified cells survive at least 1 month in the brain of mice.

### iNSCs migrate selectively to GBM

**Figure 3:**
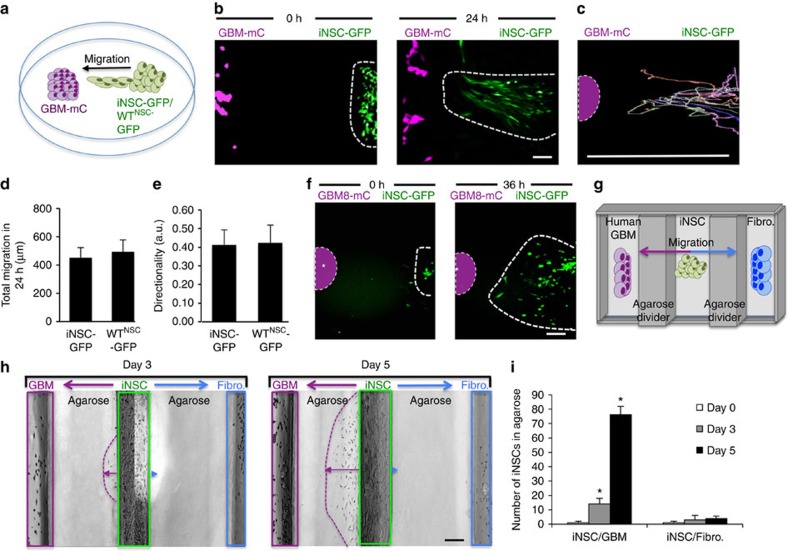
Engineered iNSCs home to GBM. (**a**) Outline of the iNSC migration assay. iNSC-GFPFLs were seeded 500 μm apart from mCherry+ human GBM cells and placed in a fluorescence incubator microscope. Time-lapse fluorescent images were captured every 10 min for 36 h and used to construct movies that revealed the migration of iNSCs in real time. (**b**) Summary images showing the migration of iNSC-GFPFL (green) towards U87-mC-FL (magenta) at 0 and 24 h after plating. (**c**) Single-cell tracings depicting the path of multiple iNSC-GFPFL migrating towards GBM cells over 24 h. Asterisk and dotted line indicate the site of GBM seeding. (**d**,**e**) Summary graph showing the total distance that iNSCs and brain-derived WT^NSC^ migrated towards GBM cells (**d**), and directionality of iNSC-GFPFL and WT^NSC^ migration to human U87 GBMs (**e**) determined from the real-time motion analysis. (**f**) Representative images showing the migration of iNSC-GFPFL to co-cultured patient-derived GBM8-mC cells (indicated by the dotted lines) at 0 and 36 h after plating. Asterisk and dotted line indicate the site of GBM8 seeding. (**g**–**i**) Outline of agarose migration assays used to determine the selectivity of iNSC migration (**g**). Three wells were created in six-well culture plates containing agarose. iNSCs were seeded in the middle well, and GBM or fibroblasts were seeded in wells on either side. Real-time imaging (**h**) and summary data (**i**) show the number of iNSCs that migrated towards the wells containing GBM cells or the fibroblasts. Scale bars in **b**,**f**,**h**, 100 μm. Scale bar in **c**, 500 μm. Data are mean±s.e.m. Data in **d**,**e**,**i** are from three independent experiments. In **d**,**e**, *P*>0.05 by Student's *t*-test. In **i**, *P<0.05 by one-way ANOVA with Tukey's multiple comparisons test.

The ability to home to solid and invasive GBM deposits is one of the most beneficial characteristics of NSC-based cancer therapies^16^,^20^. To investigate the tumouritropic nature of iNSCs, we used real-time fluorescent motion analysis of iNSC and GBM co-cultures with the migration of wild-type NSCs (WT^NSC^) as a reference (outlined in Fig. 3a). We found that iNSC-GFPFL rapidly migrated towards mCherry+ GBM cells over 24 h, covering the 500-μm gap in ∼28 h (Fig. 3b and Supplementary Movies 1–3). Single-cell tracking analysis (Fig. 3c and Supplementary Movie 2) showed that the total migratory distance of iNSCs was similar to WT^NSC^ (Fig. 3d). We next analysed the directionality of movement by determining the ratio of Euclidean distance to the accumulated distance. Straight motion was given a value of 1 and non-straight motion a value of 0. We found that iNSC migration towards co-cultured GBM cells had the same directionality as WT^NSC^ (Fig. 3e; Supplementary Movies 1 and 3). Lastly, we evaluated the capacity of iNSCs to home to patient-derived GBM cells in culture. Motion analysis showed that iNSCs seeded 500 μm from patient-derived GBM8 tumour cells migrated to the cultured tumour cells colocalizing within 36 h (Fig. 3f and Supplementary Movie 4). These results suggest that iNSCs have tumouritropic potential.

To next determine whether iNSC migration is specific for GBM cells, we designed a bi-directional under-agarose migration system to create multiple chemotactic gradients (detailed in Fig. 3g). Three wells were created in agarose 500 μm apart. GFP+ iNSCs were seeded in the middle well. To create a chemical gradient for GBM cells in one direction and fibroblasts in the opposite direction, mCherry+ GBM cells were seeded in one lateral well and BFP+ fibroblasts in the other lateral well. Real-time kinetic imaging was used to simultaneously monitor iNSC migration towards both GBM cell and fibroblasts. We found that iNSCs migrated selectively towards the GBM chemotactic signal (Fig. 3h,i). iNSC had migrated into the iNSC:GBM agarose 3 days post seeding, and the number of iNSCs as well as the migratory distance increased on day 5. In contrast, minimal iNSC migration was detected towards the fibroblast-containing wells. These findings suggest that iNSCs selectively migrate towards GBM cells.

**Figure 4:**
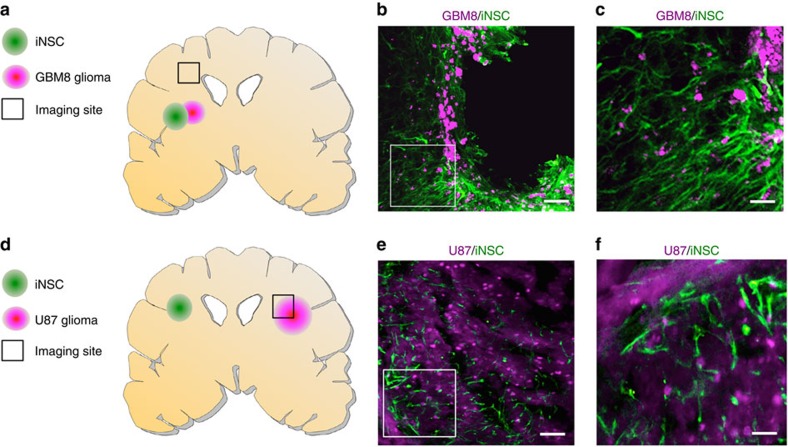
***In vivo*** **migration of iNSCs to GBMs.** (**a**–**c**) To assess iNSC tracking of invading GBM cells, GBM8-mCs were implanted into the brains of mice (*n*=7). Three days later, iNSC-GFPFLs were implanted into the tumour. Twenty-one days later, fluorescent images of cryosections were captured 1.5 mm away (indicated by the square, **a**). (**b**,**c**) Representative fluorescent images showing the colocalization of iNSC-GFPFL (green) with invading GBM8-mC (magenta; **b**), and elongated iNSC morphology (**c**). (**d**–**f**) To assess the migration of iNSCs to distant established GBMs, mCherry+ U87 were implanted into the parenchyma of mice (*n*=7). iNSC-GFPFLs were implanted into the contralateral hemisphere 5 days later. Immunofluorescent analysis of post-mortem tissue sections 21 days post implant was used to determine the presence of GFP+ iNSCs (green) at the mCherry+ GBMs (magenta; square indicates site of imaging). Scale bars in **b**,**e**, 100 and 30 μm in **c**,**f**. Data are mean±s.e.m.

To investigate iNSC migration to GBM *in vivo*, we first examined the ability of iNSCs to track GBM cells invading the parenchyma. Invasive CD133+ GBM8 cells^21^ expressing mCherry were implanted into the frontal lobe of mice (Supplementary Fig. 3a–h). Three days later, iNSC-GFPFLs were implanted at the same site as the GBM8 tumours. After 3 weeks, mice were killed and IHC was performed to determine the distribution of both GBM8 cells and iNSCs by capturing images 1.5 mm from the implantation site (summarized in Fig. 4a). We detected numerous GFP+ iNSCs colocalizing with the diffuse GBM cells as they invaded the normal brain (Fig. 4b). In addition, the iNSCs showed an elongated morphology indicative of migrating cells (Fig. 4c).

Next, we investigated the capacity of iNSCs to migrate to distant established GBMs. mCherry+ U87 tumours were implanted in mice. Five days later, iNSC-GFPFLs were implanted in the contralateral hemisphere (summarized in Fig. 4d). Immunofluorescent images of tissue sections taken 21 days post implantation showed that iNSCs colocalized with the GBM (Fig. 4e,f). Together, these observations support the conclusion that iNSCs possess tumouritropic properties and home to GBM cells *in vitro* and *in vivo*.

### Tumouricidal iNSCs are an efficacious therapy for GBM

**Figure 5:**
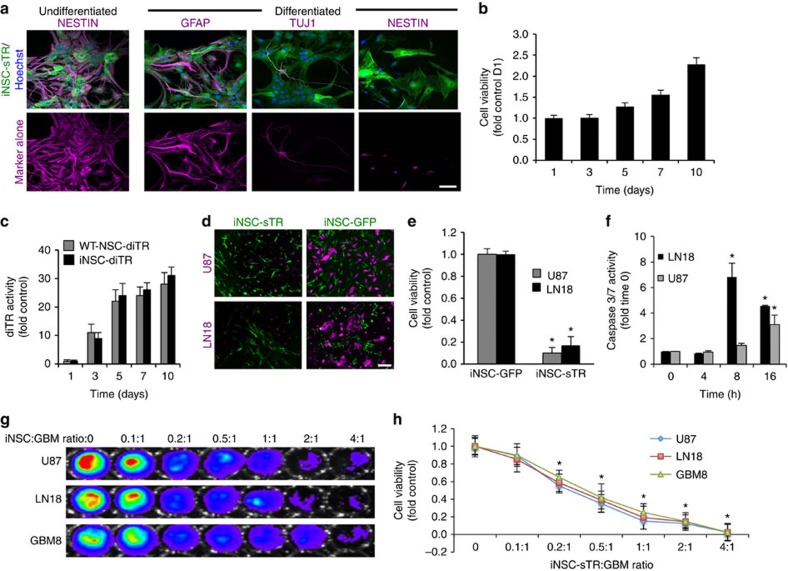
The antitumour efficacy of iNSC-sTR treatment of GBM cell lines *in vitro.* (**a**) Representative images showing the expression of nestin, GFAP or Tuj-1 in undifferentiated and differentiated iNSC-sTR cells with IHC staining (iNSC-sTR=green; staining=magenta). Magenta images alone show only the fluorescent channel corresponding to nestin, GFAP and Tuj-1 staining. (**b**) Summary graph showing the viability of iNSC-sTR over 10 days determined using bioluminescence assays. (**c**) Summary data showing the levels of diTR released by iNSC-diTR or WT^NSC^-diTR cells determined with luciferase imaging on media samples. At each time point, equal volumes of media were collected from WT^NSC^-diTR or iNSC-diTR, combined with coelenterazine, and bioluminescence imaging was performed to determine the levels of secreted diTR. (**d**,**e**) Representative images (**d**) and summary graph (**e**) showing the cell viability of U87 and LN18 human GBM cells co-cultured with iNSC-sTR or iNSC-GFP assessed using luciferase-based assay. (**f**) Summary graphs showing caspase-3/7 activity in U87 and LN18 GBM cells treated with sTR or control conditioned media determined using a bioluminescence assays that incorporates a proluminescent caspase-3/7 substrate. (**g**,**h**) Representative bioluminescence imaging (**g**) and summary data (**h**) showing the viability of U87, LN18 and GBM8 tumour cells cultured with increasing numbers of iNSC-sTR. Scale bar in **a**, 20 and 50 μm in **d**. Data are mean±s.e.m. Data in **b**,**c**,**e**,**f**,**h** are from three to four independent experiments. In **b**, *P*>0.05 by one-way ANOVA. In **c**, *P*>0.05 by repeated measures two-way ANOVA. In **e**, *P<0.05 by Student's *t*-test. In **f**,**h**, *P<0.05 by repeated measures two-way ANOVA.

To investigate the therapeutic efficacy of iNSC-based GBM treatment, we engineered iNSCs to express a secreted variant of the pro-apoptotic molecule tumour necrosis factor-α-related apoptosis-inducing ligand (TRAIL; iNSC-sTR). We have previously established the anti-GBM effects of TRAIL when delivered from engineered cell carriers^14^,^22^,^23^,^24^, making this the ideal tumouricidal molecule to characterize iNSC delivery vehicles. Robust expression of the GFP reporter was detected following transduction of the iNSCs (Supplementary Fig. 4a,b), and the modified cells could be maintained through multiple passages (Supplementary Fig. 4c). Western blot analysis confirmed that iNSCs expressed the TRAIL protein (Supplementary Fig. 4d), while quantification of TRAIL levels in the culture media showed 252 ng per 10^6^ cells over 24 h (Supplementary Fig. 4e). Immunocytochemistry showed that iNSC-sTR expressed high levels of the NSC marker nestin (Fig. 5a), while differentiation of iNSC-sTR generated GFAP+ astrocytes, Tuj-1+ neurons and reduced nestin expression (Fig. 5a). In addition, iNSC-sTR proliferated at rates similar to those observed for iNSC-GFPFL (Fig. 5b). These results suggest that modification of iNSCs with TRAIL does not interfere with their properties as stem cells.

Efficient release of tumouricidal gene products is critical to the anticancer effects of cell-based drug carriers^15^. To investigate the release of cytotoxic agents from iNSCs, we engineered iNSCs with an imageable fusion between Gaussia luciferase and TRAIL (iNSC-diTR) that we have previously used to characterize differences in TRAIL secretion between different stem cell lines^15^. Western blot analysis confirmed that the iNSCs expressed the diagnostic TRAIL (diTR) protein (Supplementary Fig. 4f). Bioluminescence imaging on media samples showed that diTR was stably released from iNSCs and levels increased with time as diTR accumulated in the culture media over 10 days (Fig. 5c). Importantly, the levels of diTR released by iNSCs was similar to those released by WT^NSC^ that are proven to effectively eradicate GBM^14^,^15^,^16^,^18^.

To evaluate the anti-GBM efficacy of cytotoxic iNSCs, iNSC-sTRs or control iNSC-GFPs were co-cultured with human U87 or LN18 GBM cells expressing mCherry and firefly luciferase (mC-FL; Fig. 5d). Luciferase assays and fluorescence imaging revealed that iNSC-sTR decreased the viability of U87 and LN18 human GBM cells to 93% and 87%, respectively (Fig. 5e). It is well established that TRAIL-induced cell death occurs via caspase-mediated apoptosis^25^, and treatment of U87 and LN18 GBM lines with conditioned media from iNSC-sTR treatment significantly upregulated caspase-3/7 activity over time (Fig. 5f). We detected a decline in caspase activity in LN18 cells at 16 h that was likely due to an extensive cell loss at this time point caused by robust TRAIL-induced cell death. Next, we cultured U87, LN18 and GBM8 cells with increasing numbers of iNSC-sTR to determine the ratio of iNSC-sTRs:GBMs that induced tumour cell death. Using cell viability assays, we detected statistically significant reductions in tumour viability at ratios as low as 0.2:1, and GBM cell death increased with increasing numbers of iNSC-sTR (Fig. 5g,h).

**Figure 6:**
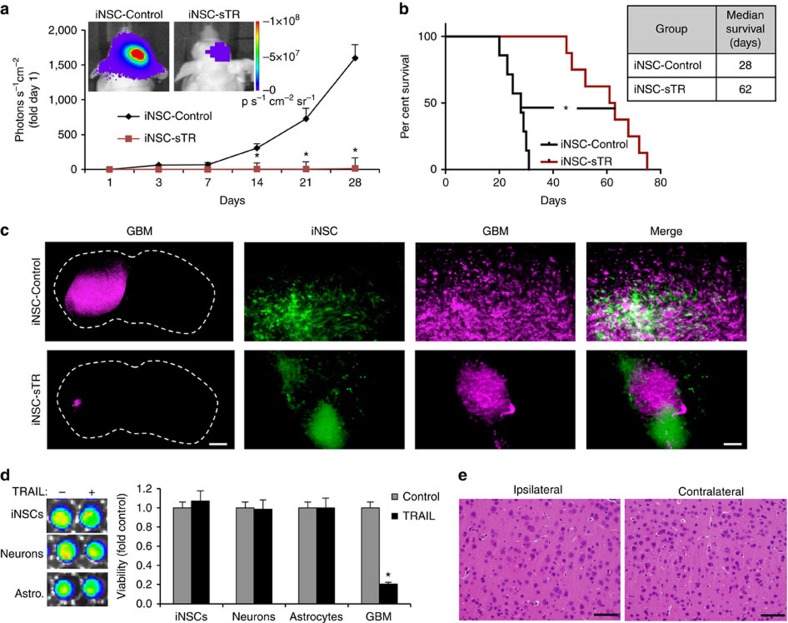
Antitumour efficacy of iNSC-sTR treatment of orthotopic human GBM xenografts. Human U87 GBM cells expressing mCherry and firefly luciferase were implanted into the parenchyma of mice with iNSC-sTR or control iNSC-GFP. (**a**) Serial bioluminescence imaging showing the growth of the human GBM xenografts treated with iNSC-sTR or control iNSC-GFP through 28 days (*n*=12 per group). (**b**) Kaplan–Meier curves showing the survival of U87-bearing animals treated with iNSC-sTR or control iNSC (*n*=12 per group). (**c**) Representative fluorescent micrographs of brain sections from control- or iNSC-sTR-treated mice bearing mC-FL-expressing U87 tumours. Magenta=U87; Green=iNSC-sTR or iNSC-GFP. (**d**) Images and summary data showing the viability of iNSCs, neurons, astrocytes and U87 GBM cells cultured with conditioned media from iNSC-sTR or iNSC-GFP. (**e**) Representative histological images of the brain sections from mice injected intracranially with iNSC-sTR (*n*=5). Images were captured 0.5 mm from the implantation site (ipsilateral) and the same distance from the midline in the opposite hemisphere (contralateral). Scale bar in **c**, 1,000 and 30 μm in magnification. Scale bar **e**, 30 μm. Data are mean±s.e.m. Data in **a**,**b**,**d** are from three independent experiments. Data in **a**,**b** are *n*=12 per group. In **a**, *P<0.05 by repeated measures two-way ANOVA. In **b**, *P<0.01 by exact log-rank test. In **d**, *P<0.01 by Student's *t*-test.

To test the *in vivo* efficacy of iNSC-based therapy, we first determined the effects of iNSC-sTR treatment on solitary human GBMs. Human U87 GBM cells expressing mC-FL were implanted intracranially with iNSC-sTR or control iNSC-GFP, and tumour volumes were followed using serial bioluminescence imaging. We found that iNSC-sTR treatment induced a marked reduction in tumour growth and decreased GBM volumes 123-fold by day 28 (Fig. 6a). In addition, iNSC-sTR-treated animals survived more than 2 months before succumbing to recurrent tumours (median survival: 62 days; Supplementary Fig. 5), while control animal succumbed to GBM growth in only 28 days (Fig. 6b). To verify the changes in GBM volumes detected by bioluminescence imaging, fluorescence imaging of post-mortem brain sections collected from control and iNSC-sTR-treated animals 21 days after treatment confirmed the presence of large mCherry+ GBM in the control-treated animals that encompassed the majority of the hemisphere where the cells were implanted (Fig. 6c). In contrast, a small GBM was detected in the iNSC-sTR-treated animals near the initial site of implantation. In these tissue sections, GFP+ iNSC-GFP and iNSC-sTR were distributed around both the control and sTR-treated tumours 21 days post injection.

Next, we investigated whether the cytotoxic effect of iNSC-sTR was specific to cancer cells. Cultured iNSCs, neurons, astrocytes and GBM cells were treated with conditioned media from iNSC-sTR or iNSC-GFP. Cell viability assays performed 48 h later showed that viability was not reduced in iNSCs, neurons or astrocytes treated with iNSC-sTR media, although GBM viability was still significantly reduced by the same conditioned media (Fig. 6d). To investigate iNSC-sTR toxicity *in vivo*, we implanted iNSC-sTR into the brain of non-tumour-bearing Nude mice. Pathologic analysis of tissue sections 2 weeks after implantation showed no overt signs of toxicity in the brain. Neural content and cell morphology were similar at sites 0.5 mm from the site of iNSC-sTR injection and the same distance from the midline in the contralateral hemisphere (Fig. 6e).

**Figure 7:**
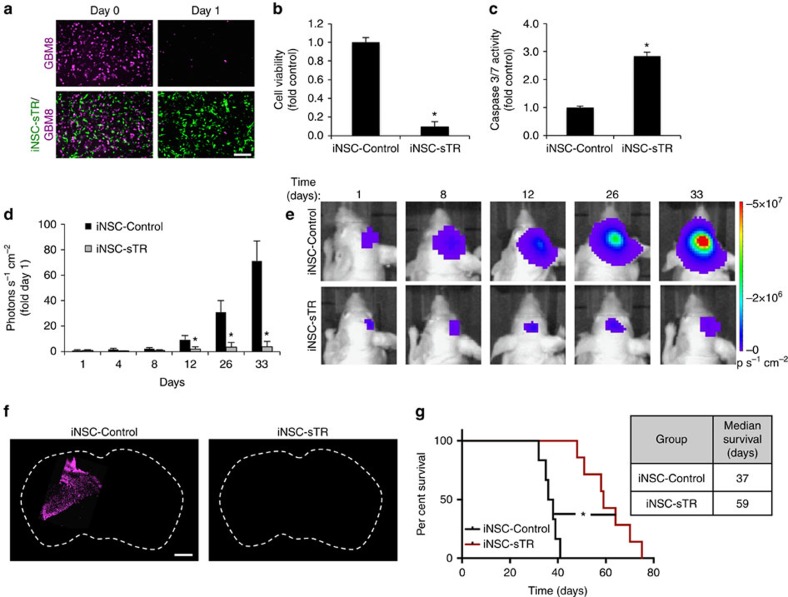
Efficacy of iNSC-sTR therapy for highly invasive patient-derived GBM8 xenografts. (**a**) Representative images of patient-derived GBM8 cancer cells expressing mC-FL (magenta) co-cultured with iNSC-sTR (green). (**b**) Summary data showing the cell viability of GBM8 cells after 24 h of co-culture with iNSC-sTR or control iNSCs assessed by luciferase assay. (**c**) mC-FL-expressing GBM8 glioma cells were incubated with conditioned media from iNSC-sTR or control cells, and caspase-3/7 activity was determined 18 h later. (**d**–**g**) Highly invasive patient-derived GBM8 cells expressing mC-FL were implanted into the parenchyma of mice. Three days later, iNSC-sTRs or iNSC-GFPs were implanted into tumour. (**d**,**e**) Serial bioluminescence images tumour growth in iNSC-sTR- or iNSC-GFP-treated animals (*n*=12 per group). (**f**) Representative fluorescent micrographs of the brain sections from control- or iNSC-sTR-treated mice bearing mC-FL-expressing GBM8 tumours (magenta). (**g**) Kaplan–meier curves revealing the survival in animals treated with iNSC-sTR or iNSC-GFP (*n*=12 per group). Scale bar in **a**, 50 and 1,000 μm in **f**. Data are mean±s.e.m. Data in **b**–**e** are from three independent experiments. In **b**,**c**, *P<0.05 by Student's *t*-test. Data in **d**,**g** are *n*=12 per group. In **b**,**c,** *P<0.05 by paired *t*-test. In **d**, *P<0.05 by repeated measures two-way ANOVA. In **g**, *P<0.01 by exact log-rank test.

To determine the efficacy of iNSC-sTR treatment on patient-derived human GBM cells, we co-cultured GBM8 cells expressing mC-FL (GBM8-mC-FL) with iNSC-sTR or control iNSC-GFP. Cell viability assays showed that iNSC-sTR decreased GBM8 viability that was accompanied by a parallel increase in caspase activation (Fig. 7a–c). The patient-derived GBM8 cells are highly invasive and form diffuse tumours *in vivo*^21^. We used this model to define the efficacy of iNSC-sTR therapy on established diffuse GBMs by implanting GBM8-mC-FL cells into the mouse brain parenchyma. Three days later, iNSC-sTR or control cells were administered directly into the established tumours. Serial bioluminescence imaging showed that iNSC-sTR treatment attenuated the progression of GBM8 tumours, reducing tumour burden by 18.3-fold compared with control 33 days after treatment (Fig. 7d,e). These imaging results were confirmed by post-mortem IHC that showed a small GBM present after iNSC-sTR therapy compared with control treatment (Fig. 7f). iNSC-sTR therapy also led to a significant extension in survival as iNSC-sTR-treated animals survived an average of 59 days compared with only 37 days in control-treated mice (Fig. 7g), yet the animals succumbed to eventual tumour recurrence (Supplementary Fig. 3i). Together, these results show that iNSC-sTR therapy has significant therapeutic effects against malignant and invasive GBM, and markedly prolongs the survival of tumour-bearing mice.

**Figure 8:**
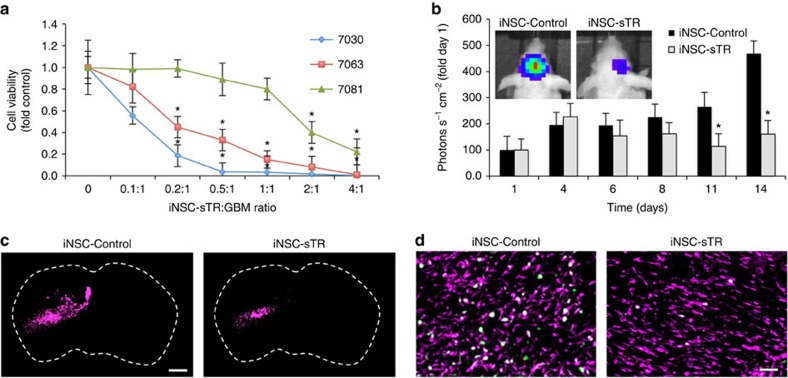
iNSC-sTR treatment of patient-derived GBMs. (**a**) Summary data of the patient-derived cancer cells lines 7030, 7081, 7063, GBM6 treated with increasing volumes of conditioned media from iNSC-sTR or control iNSC-GFP cells. Cell viability was assessed 24 h after treatment. (**b**) Representative bioluminescent images and summary data showing the progression of 7063 xenografts treated with iNSC-sTR or iNSC-GFP (*n*=12 per group). (**c**) Representative fluorescent micrographs of the brain sections from control- or iNSC-sTR-treated mice bearing 7063 GBM xenografts (magenta). (**d**) Ki-67 (green) staining of post-mortem tissue sections 7063 (magenta) tumours treated with iNSC-control or iNSC-sTR. Colocalization of the signal is shown in white. Scale bar in **c**, 1,000 and 100 μm in **d**. Data in **a** are from three independent experiments. Data in **b** are *n*=12 per group. In **a**,**b**, *P<0.05 by repeated measures two-way ANOVA.

GBM is a heterogeneous tumour type, and response to therapy can vary. As such, we further validated the efficacy of iNSC-sTR therapy against additional patient-derived GBM cell lines. Three different human patient-derived GBM cell lines were cultured with increasing numbers of iNSC-sTR. Cell viability assays performed 24 h post treatment revealed that iNSC-sTR reduced the viability of all three GBM lines, although the response varied (Fig. 8a). The 7030 line showed the greatest sensitivity to TRAIL, 7081 showed the greatest resistance, while the 7063 showed intermediate sensitivity, with significant cell death occurring at an iNSC-sTR:GBM ratio as low as 0.2:1. To investigate the iNSC-sTR therapy against additional patient-derived GBMs *in vivo*, CD133+ 7063 GBM cells expressing mCherry and FLuc were xenografted into mice (Supplementary Fig. 6). Seven days later, iNSC-sTRs or iNSC-GFPs were implanted into the established tumours. Serial bioluminescence imaging showed that iNSC-sTR treatment significantly inhibited the growth of the GBM compared with control-treated animals (Fig. 8b). Analysis of post-mortem tissue sections confirmed the reduction in tumour volumes by iNSC-sTR treatment (Fig. 8c) and a reduction in the number of proliferating tumour cells (Fig. 8d). These results support the conclusion that iNSC-sTR treatment has efficacious effects against patient-derived GBMs.

## Discussion

In this study we investigated the use of iNSCs as cellular delivery vehicles for GBM therapy. We show that iNSCs can be engineered with therapeutic or diagnostic transgenes without affecting their ability to proliferate or differentiate. We found that iNSCs rapidly migrate to GBM cells in culture and home to human GBM xenografts *in vivo*. We also observed that iNSCs stably secrete the anticancer protein TRAIL and kill human GBM cells in culture. *In vivo*, iNSC-sTR therapy markedly suppressed tumour growth and significantly extended the survival of mice bearing malignant or invasive human GBM xenografts. Together, these data support the potential of iNSCs to serve as highly effective drug-delivery vehicles for treatment of solid and invasive brain tumours.

Cellular reprogramming is an exciting technology that has offered new opportunities for developing large numbers of personalized stem cells^3^. iNSC technology represents a promising alternative to iPSCs and creates cells that are ideal for transplant. To date, iNSCs have been generated through two main methods: transient expression of reprogramming factors or forced expression of NSC-specific transcription factors. In both cases, the resulting iNSCs gave rise to astrocytes, neurons and oligodendrocytes. In addition, three studies have confirmed the lack of tumorigenesis following iNSC implantation *in vivo*^6^,^7^,^8^,^10^. These data clearly suggest the beneficial properties of iNSCs for therapy; yet, no studies have explored the efficacy of iNSC-based treatment for diseases of the CNS. While iNSCs will undoubtedly be explored for treatment of neurological disorders, iNSCs could potentially benefit human health through application in other fields that include the development of more efficient drug-delivery vehicles. Cancers of the CNS are a devastating disease^11^, and iNSC technology could fill the critical need for autologous drug carriers. The *in vitro* and *in vivo* findings in this study demonstrate the feasibility of iNSC-based therapy for GBM and form a foundation to justify continued development of iNSC-based treatments.

We investigated the effects of engineering iNSCS with LVs encoding optical reporters and anticancer therapeutic transgenes on cell fate. We previously demonstrated the ability to engineer human and mouse mesenchymal and NSCs to allow *in vivo* tracking as well as GBM killing^13^,^14^,^15^,^24^. We found that iNSCs engineered with GFPFL or TRAIL proliferated and differentiated with the same efficiency as unmodified cells. In addition, the modified iNSCs stably secreted the pro-apoptotic TRAIL at levels that were equivalent to WT^NSC^. Therefore, it is likely that iNSCs will stably release a variety of antitumour transgenes similar to WT^NSC^ (ref. 26). This would allow a broad application of iNSC-based cancer therapy through creation of a panel of therapeutic iNSCs to target tumour cells regardless of the underlying genetic drivers or molecular profile. It could also allow therapeutic iNSCs to be engineered with clinically relevant imaging molecules, such as PET-compatible cytosine deaminase or thymidine kinase, to track the fate of cells once they enter patient trials.

We found that iNSCs possess the same unique tumour-homing capacity as WT^NSC^. Unlike traditional Boyden chamber assays, we used real-time fluorescence motion analysis to generate time-lapse movies and quantified the migratory velocity, distance and directionality of both iNSC and WT^NSC^ to co-cultured human GBM cells. We discovered that iNSCs home selectively to GBM cells at a velocity and directionality equivalent to WT^NSC^. *In vivo*, we found that iNSCs colocalized with diffuse GBM cells 1.5 mm from the primary tumour site 3 weeks after implantation and colocalize with solid GBMs implanted in the contralateral hemisphere of mice. The tumour-homing properties are one of the most important features of NSC-based GBM therapy. WT^NSC^ are known to migrate through the brain to both solid and diffuse GBM cells with greater speed than mesenchymal stem cell carriers^27^. GBM therapy is more effective when delivered from migratory NSCs than non-migratory fibroblasts^20^. Extensive studies have uncovered the molecular mechanisms mediating WT^NSC^ homing to GBM cells (summarized in refs 28, 29). WT^NSC^ tumouritropic homing is known to occur by chemotaxis and several key signalling pathways have been identified, such as the CXCR4/SDF-1 signalling axis^29^,^30^,^31^,^32^,^33^,^34^. These key pathways are upregulated in WT^NSC^ but not on normal cells (that is, fibroblasts and astrocytes). Thus, fibroblasts do not have the same tumour-homing capacity that NSCs possess. We demonstrate for the first time that iNSCs have the same tumour-homing capacity as brain-derived NSCs. This suggests that these tumour-homing pathways are upregulated as fibroblasts are converted into iNSCs. This adds tumouritropic signalling pathways to the list of features both iNSCs and brain-derived NSC share, but fibroblasts do not. Other features shared by both iNSCs and NSCs (but not fibroblasts) include nestin expression, Pax6 expression, the ability to differentiate into astrocytes, neurons and oligodendrocytes, and so on^4^,^35^. We are exploring the expression of multiple chemotactic pathways by iNSC cells, using short interfering RNA-mediated knockdown to investigate the relative contribution of each pathway in *in vitro* and *in vivo* model systems, and performing side-by-side comparison with brain-derived NSCs. Future studies will be required to determine whether iNSCs are able to migrate to areas of stroke or other brain lesion similar to WT^NSC^.

We found that iNSC-sTR inhibited the growth of malignant and patient-derived xenografts, as well as increased the survival of tumour-bearing mice. The effects on tumour progression and survival were greater in the U87 model than in the GBM8 model. We have previously observed similar findings using WT^NSC^-based delivery of TRAIL^13^,^15^,^23^. This is likely due to the invasive nature of the GBM8 cells that, similar to diffuse GBM in patients, allows them to evade treatment. In addition, we found that animals eventually succumbed to GBM recurrence despite the initial reductions in tumour volumes. This suggests that multiple rounds of iNSC-sTR therapy may be required to eradicate the tumours or sustain GBM suppression. We previously demonstrated that combination strategies improve TRAIL killing and overcome resistant tumour populations^13^,^14^, supporting the development of iNSC-based combination therapies.

The potential for autologous NSC-based therapy is one of the key benefits to iNSC cell carriers. Testing autologous iNSC-based therapies for GBM in mice could be accomplished but would require a syngeneic mouse model. Few studies have explored the efficacy of NSC-based therapy using mouse-derived GBMs in C57BL/6 mice. This is due in part to the lack of clinically relevant models^36^. We performed these initial proof-of-concept studies using human xenograft models in immune-deficient mice. Although this does not uncover the benefits of autologous transplant, our findings clearly demonstrate that iNSC-based therapy has a significant impact on the growth of human GBM xenografts. These feasibility studies can now be followed with more extensive investigation where iNSC carriers are created and used for truly autologous therapy in mouse models of murine-derived orthotopic allograft. We envision an eventual clinical approach where skin biopsies from GBM patients could be rapidly converted to iNSCs that are engineered with antitumour therapies and re-implanted into the patient for autologous cell-based GBM therapy.

Although our results are promising, there are limitations. One of the greatest limitations to transplant therapy with stem cells created by cellular reprogramming technology has been the use of viral vectors. As such, a variety of studies have demonstrated the potential for generating iPSCs through non-viral methods^37^, and small molecules or RNA-based methods are particularly attractive. Recently, Adler *et al.* used a nanoparticle-based approach to successfully convert fibroblasts directly into neurons^38^. In addition, advancement towards clinical iNSC-based carriers will require the development of human iNSC (h-iNSC) cell carriers. In a subset of experiments, Ring *et al.* showed that their approach of Sox2 transduction and culturing on feeder cells could convert human fibroblasts into h-iNSCs that generated astrocytes, neurons and oligodendrocytes *in vitro* and did not form tumours *in vivo*. Matsui *et al.* reported that generation of h-iNSCs could also be accomplished by partial reprogramming of cells using Yamanaka factors. The continued development and characterization of h-iNSC cells will pave the way for future investigations into the efficacy of h-iNSC therapies for CNS disorders. Once cytotoxic h-iNSCs are generated, they will need to be tested against the HB1.F3.CD cell line. This human NSC is an established line that has brought the field of NSC-based therapy for GBM into human patient testing. The line has been extensively characterized to gain approval for first-in-human testing^17^,^18^. Yet, one potential limitation of this cell lines is that it requires allogeneic transplant. In contrast, the autologous h-iNSC approach has the potential to avoid immune rejection, increase carrier persistence and improve therapeutic efficacy. Side-by-side comparisons between HB1.F3.CD and cytotoxic h-iNSCs will be critical to reveal differences in migratory rate, safety, drug release and tumour killing. The benefits of autologous versus allogeneic therapy will require large animal testing, and may not be fully revealed until human testing is approved.

In summary, our results provide the first evidence that iNSC can treat diseases of the CNS and demonstrate the feasibility and efficacy of iNSC-based therapy for GBM. We found that iNSCs migrate to GBM, secrete anticancer molecules and regress GBM with the same efficiency as WT^NSC^ drug carriers. Stem cell-based therapy for GBM has recently entered clinical trials for primary and recurrent GBM, and trials for breast cancer and neuroblastoma will be launched soon. With continued development, iNSC technology will have an impact on the design of these trials as a viable alternative drug-delivery vehicle with the potential for autologous treatment.

## Methods

### Cell lines

U87 human GBM cells and human astrocytes were purchased from American Type Culture Collection (ATCC, Manassas, VA). WT^NSC^ were purchased from StemCell Technologies (Vancouver, Canada). GBM8s were a kind gift from Dr Hiroaki Wakimoto (Massachusetts General Hospital, Boston, MA). 7030, 7081, 7063 and GBM6 were a kind gift from Dr William Weiss (University of California, San Francisco Cancer Center). 293T/17 cells were purchased from the UNC Tissue Culture Facility. All lines were grown as previously described^15^,^39^,^40^, authenticated and tested for mycoplasma. Reprogramming LVs were purchased from Addgene: TetO-FUW-Sox2 (deposited by Dr Rudolph Jaenisch), Tet-O-FUW-Brn2 (deposited by Dr Marius Wernig), Tet-O-FUW-FoxG1 (deposited by Dr Marius Wernig) and FUW-M2rtTA (deposited by Dr Rudolph Jaenisch). All cDNAs were under the control of the tetracycline promoter. Mouse iNSCs were generated following the method of Lujan *et al.*^6^. Overall, 200,000 mouse embryonic fibroblasts (MEFs; a kind gift from Dr Larysa Pevny, UNC) were seeded in six-well plates coated with poly-L ornithine. The MEFs were isolated from C57BL/6J mice at E13.5 and utilized at passage number 3. MEFs were transduced with the LV cocktail containing rTTA, and Sox2, FoxG1 and Brn2 under the control of the tetracycline-inducible promoter in media containing polybrene (5 μg ml^−1^, Sigma). Two days after infection, the media was changed to N3 media containing fibroblast growth factor (FGF; 10 ng ml^−1^, Peprotech), epidermal growth factor (EGF; 10 ng ml^−1^, Peprotech) and doxycycline (10 μg ml^−1^, Sigma)^6^. Media was changed every 3 days. Neurosphere formation was induced by culturing in low-adherent flasks (Corning, Tewksbury, MA). Karyotype analysis was performed using KaryoLogic (Research Triangle Park, NC).

### Lentiviral vectors

In addition to the reprogramming vectors described above, the following LVs were used in this study: LV-GFP-FL, LV-GFP-RLuc, LV-mC-FL, LV-sTR and LV-diTR. GFP-RLuc and GFP-FL were constructed by amplifying the cDNA encoding Renilla luciferase or firefly luciferase using the vectors luciferase-pcDNA3 and pAC-hRluc (Addgene), respectively. Restriction sites were incorporated in the primers, the resulting fragment was digested with BglII and SalI and was ligated directionally using BglII/SalI-digested pEGFP-C1 (Clontech). The GFP-FL or GFP-RLuc fragments were digested with AgeI (blunted) and SalI, ligated into pTK402 (provided by Dr Tal Kafri, UNC Gene Therapy Center) and then digested with BamHI (blunted) and XhoI to create LV-GFP-FL or LV-GFP-RLuc. Similarly, mC-FL was created by amplifying the cDNA encoding firefly luciferase from luciferase-pcDNA3, ligating into BglII/SalI-digested mCherry-C1 (Clontech) and ligating the mC-FL fragment into the pTK402 LV backbone using blunt/XhoI sites. To create LV-sTR and LV-diTR, the cDNA sequence encoding sTR or diTR was PCR-amplified using custom-synthesized oligonucleotide templates (Invitrogen). The restriction sites were incorporated into the primers, the resulting fragment was digested with BamH1 and XhoI and was ligated in-frame into BamH1/XhoI-digested pLVX plasmid (a kind gift from Dr Scott Magness, UNC Department of Medicine). Both LV-sTR and LV-diTR have IRES-GFP (internal ribosomal entry site-GFP) elements in the backbone as well as in cytomegalovirus-driven puromycin element. All LV constructs were packaged as LV vectors in 293T/17 cells using a helper virus-free packaging system as described previously^41^. iNSCs, GBM cells and WT^NSC^ were transduced with LVs at varying multiplicity of infection by incubating virions in a culture medium containing 5 μg ml^−1^ polybrene (Sigma), and the cells were visualized for fluorescent protein expression using fluorescence microscopy.

### Cell viability and passage number

To assess the proliferation and passage number of modified and unmodified iNSCs, iNSCs expressing GFP-FL, sTR or unmodified cells were seeded in 96-well plates. Cell viability was assessed over a period of 10 days using the CellTiter-Glo luminescent cell viability kit (Promega). Maximum passage number was assessed by monitoring the number of times iNSCs, iNSC-sTR or WT^NSC^ were subcultured without alterations in morphology, growth rate or transduction efficiency.

### *In vitro* differentiation and IHC

To determine the effects of LV modification on iNSC differentiation, iNSCs were transduced with LV-GFP-FL or LV-sTR. Engineered or unmodified cells were fixed, permeabilized and incubated with anti-nestin monoclonal antibody (Millipore, MAB353, 1:250) or anti-Sox2 (Abcam, AB97959, 1:200). Cells were washed and incubated with mouse anti-goat CF555 secondary antibody (Biotium, 20031, 1:1,000) for 1 h. Cells were then washed, mounted and imaged using an Olympus FV 1200 confocal inverted microscope. For differentiation, engineered or non-transduced iNSCs were cultured for 12 days in N3 media depleted of doxycycline, EGF and FGF. Cells were then stained with antibodies directed against nestin, GFAP (Millipore, MAB3405, 1:250) or Tuj-1 (Sigma, T8578, 1:1,000) and were detected with CF555 secondary antibodies (Biotium, 20031, 1:1,000). To determine the percentage of differentiated iNSCs expressing GFP-FL or sTR compared with unmodified cells, differentiated cells were additionally incubated with Hoechst 33253 (Sigma-Aldrich, 861405, 10 μg ml^−1^) to stain nuclei. The number of differentiated cells was determined by manually counting positively stained cells determined in at least three nonoverlapping fields and expressed as a percentage of the total cell determined by counting Hoechst-positive nuclei. To determine CD133 expression in GBM cells, GBM8 or 7063 tumour cells were fixed, permeabilized and incubated with anti-CD133 monoclonal antibody (Millipore, MAB4399, 1:100).

### Tissue processing

Immediately after the last imaging session, mice were killed, perfused with formalin and brains extracted. The tissue was immediately immersed in formalin. Coronal sections (30 μm) were generated using a Leica CM1850 cryostat (Leica Biosystems, Buffalo Grove, IL). The sections were incubated for 1 h in a blocking solution (0.3% bovine serum albumin (BSA), 8% goat serum and 0.3% Triton X-100) at room temperature, followed by incubation at 4 °C overnight with the following primary antibodies diluted in blocking solution: (1) anti-nestin (Millipore, MAB353, 1:250), (2) anti-GFAP (Millipore, MAB3405, 1:250), (3) anti-Tuj-1 (Sigma, T8578, 1:1,000), (4) CD133 (Millipore, MAB4399, 1:100) and (5) Ki-67 (Dako, M7240, 1:20). The sections were washed three times with PBS, incubated in the appropriate secondary antibody and visualized using a confocal microscope (Olympus). Paraffin embedding, tissue sectioning and haematotoxylin and eosine staining were performed by the UNC Animal Histopathology Core.

### iNSC fate and toxicity studies

To determine the survival of iNSCs in mice, iNSCs expressing GFP-FL (7.5 × 10^5^ cells per mouse) were suspended in PBS and implanted stereotaxically in mice (*n*=10 per group) in the right frontal lobe 2 mm lateral from the bregma and 2 mm deep. iNSC survival was determined by bioluminescence imaging of mice performed every 7 days for 4 weeks as described previously^13^,^15^,^42^.

To determine the fate of iNSCs at a cellular resolution, animals were killed 21 days post implantation and brains were extracted and sectioned. Tissue sections were stained with antibodies against nestin, GFAP and Tuj-1, and visualized using a secondary antibody labelled with CF555, as is described previously. In a subset of animals, iNSC-GFPFLs (7.5 × 10^5^ cells per mouse) were mixed with human U87 GBM cells expressing mCherry (5 × 10^4^ cells per mouse; *n*=10).

To determine the toxicity of iNSC-sTR in non-tumour brain, iNSC-sTRs (7.5 × 10^5^ cells per mouse) were stereotactically injected into the parenchyma of Nude mice (*n*=5). Fourteen days later, mice were killed, and their brains were harvested, embedded in paraffin, sectioned and stained with haematotoxylin and eosine. The tissue sections were analysed by Dr Ryan Miller (Department of Pathology, UNC Hospitals). Images were captured 0.5 mm from the glial scar induced by the needle tract, and the same distance from the midline in the contralateral (non-stem cell) hemisphere.

### Real-time imaging and motion analysis

WT^NSC^ and iNSCs expressing GFP were seeded in microculture inserts in glass bottom microwell dishes (MatTek, Ashland, MA) using two-chamber cell culture inserts (Ibidi, Verona, WI). U87 glioma cells expressing mCherry were plated into the adjacent well (0.5 mm separation) or the well was left empty (control). Twenty-four hours after plating, cells were placed in a VivaView live cell imaging system (Olympus) and were allowed to equilibrate. The insert was removed and cells were imaged at × 20 magnification every 10 min for 36 h in six locations per well (to monitor sufficient cell numbers) in three independent experiments. NIH Image was used to generate movies and determine the migrational total distance migrated and the directionality of migration using the ‘Manual Tracking' and ‘Chemotaxis Tool' plugins.

To determine directional migration, we used an under-agarose cell migration assay to create multiple chemotactic gradients^43^. A 0.6% agarose solution was prepared, and 3 ml was added to each well of six-well culture plates and allowed to solidify. A 2-ml aspirating pipette attached to a vacuum was used to create three cavities in the agarose ∼500 μm apart. iNSCs were seeded in the central well, U87 GBM cells were seeded in one lateral well and fibroblasts were seeded in the remaining lateral well. differential interference contrast images were captured 0, 3 and 5 days post seeding to visualize cell migration. iNSC migration was quantified by manually counting iNSCs present in the iNSC:GBM agarose or iNSC:fibroblast agarose from multiple visual fields 0, 3 and 5 days post seeding.

### iNSC migration *in vivo*

To determine the migration of iNSC to invasive GBM, mice (*n*=7) were implanted with GBM8 cells expressing mC-FL (5 × 10^5^ cells per mouse). Three days later, iNSC-GFP-FL (7.5 × 10^5^ cells per mouse) were implanted into the same implantation site as the GBM8. To assess migration, animals were killed at 14, 21 and 28 days post implantation. Their brains were processed as described below. Images were captured showing the distribution of red GBM8 tumour cells and green iNSCs 1–2 mm from the initial site of implantation adjacent to the lateral ventricle. To determine the migration of iNSCs to established GBMs, U87-mC-RLs were implanted in mice (5 × 10^4^ cells per mouse, *n*=7). Five days later, iNSC-GFPFLs were implanted in the contralateral hemisphere 2 mm from the midline. Mice were killed 21 days later and immunofluorescent analysis of post-mortem tissue sections was used to visualize the colocalization of GFP+ iNSC with the mCherry+ GBM.

### *In vitro* toxicity assays

To determine the toxicity of iNSC-sTR against multiple cell types, iNSCs, human neurons (a kind gift from Dr Anne Taylor, UNC Neuroscience), human astrocytes (ATCC) and U87 GBM cells (5 × 10^3^ cells per well) were seeded in 96-well plates. Conditioned media collected from iNSC-sTR or control iNSC-GFP were added to each well. Cell viability was determined 48 h later using the CellTiter-Glo luminescent assay.

### *In vitro* imaging of diTR secretion

The culture medium containing secreted fusion proteins or the cells were collected from iNSC-diTR or WT^NSC^-diTR 24 h after refreshing. The luciferase activity in the medium was determined by incubating the medium or cells with 1 μg ml^−1^ coelenterazine and was imaged in a luminometer as described^15^.

### iNSC antitumour efficacy

To determine the therapeutic efficacy of iNSC-sTR against solid human U87 tumours, a combination of iNSC-sTR or iNSC-GFP-RLuc (7.5 × 10^5^ cells per mouse) was stereotactically implanted into the right frontal lobe of mice (*n*=12 per group) together with U87-mC-FL cells (1 × 10^6^ cells per mouse). Therapeutic response was then determined by following tumour volumes with FL bioluminescence imaging as described previously^15^,^42^. Briefly, mice were given an intraperitoneal injection of D-Luciferin (150 μl of 4.5 mg ml^−1^ in saline solution per mouse) and photon emission was determined 5 min later using an IVIS Kinetic Optical System (PerkinElmer) with a 5-min acquisition time. Images were processed and photon emission quantified using the LivingImage software (PerkinElmer). In addition, mice were followed for survival over time.

To investigate the efficacy of iNSC-sTR against invasive patient-derived human GBM cells, mice were stereotactically implanted in the right frontal lobe with GBM8 or 7063 GBM cells expressing mC-FL (5 × 10^5^ cells per mouse). Three days later, iNSC-sTR (*n*=12, 7.5 × 10^5^ cells per mouse) or iNSC-GFP-RLuc (*n*=12, 7.5 × 10^5^ cells per mouse) were implanted into the tumour implantation site. Changes in tumour volume were assessed by FL imaging as described above and mice were followed for survival over time.

Female Nude mice 4–6 weeks of age were used in all experiments and purchased from the UNC Animal Studies Core. Mice were bred in-house by the core. All experimental protocols were approved by the Animal Care and Use Committees at The University of North Carolina at Chapel Hill, and care of the mice was in accordance with the standards set forth by the National Institutes of Health Guide for the Care and Use of Laboratory Animals, USDA regulations, and the American Veterinary Medical Association.

### Cytogenetics

Cytogenetic analysis was performed by Karyologic (Research Triangle Park), and a total of 20 G-banded metaphase spreads were analysed. Banding technique used was G-bands by trypsin using Wright stain (GTW) and banding resolution was reported as ‘good'. Analysis reported karyotype as ‘normal'.

### Western blot analysis

iNSCs expressing sTR or diTR or control iNSCs were lysed with RIPA buffer containing a protease cocktail (Roche, Basel, Switzerland) and centrifuged at 30,000*g* for 30 min at 4 °C. Equal amounts of total cell protein (1, 5, 10 μg) were denatured, separated using SDS–PAGE and transferred to nitrocellulose membrane, blocked with 5% milk and incubated for 1 h at room temperature with rabbit polyclonal antibodies to TRAIL (ProSci, Poway, CA, 113, 1 μg ml^−1^)^15^. Blots were developed using enhanced chemiluminescence reagents (Amersham). Membranes were then exposed to film for 30 s to 30 min. Images shown in Supplementary Fig. 4 have been cropped for presentation. Full-size images are presented in Supplementary Fig. 7.

### Enzyme-linked immunosorbent assay to determine sTR secretion

The levels of sTR secretion from iNSCs was determined by measuring sTR levels in conditioned media with the TRAIL Immunoassay Kit (Biosource International, Camarillo, CA) following the manufacturer's protocols. Recombinant human TRAIL provided in the assay kit was used as a standard.

### Flow cytometric analysis

GBM8 cells expressing EGFP (1 × 10^7^ cells) were washed in PBS and were resuspended in 100 μl of buffer composed of MACS BSA solution (Miltenyi Biotec, 130-091-376) diluted 1:20 in Auto-MACS Rinsing Solution (Miltenyi Biotec, 130-091-222). Ten microlitres of the anti-CD133 antibody conjugated to phycoerythrin (PE; Miltenyi Biotec, 130-090-853, 1:100) was added and the cells were incubated in the dark at 4 °C for 10 min. GBM8 cells were incubated without the antibody and were processed in the same way for use as the control. After 10 min of incubation, cells were washed using 1 ml of buffer and centrifuged at 300*g* for 10 min. The supernatant was aspirated and the cell pellet was resuspended in 1 ml buffer. The bench top acquisition was conducted on a Sony SH800 FACS instrument. Forward Scatter (FSC-area) and Back Scatter (BSC-area) gates were set to exclude debris and double discrimination gates (FSC-area versus FSC-height) were set to exclude multimers. Single colour controls were used to compensate for spectral overlap between EGFP and PE. Negative cell gates were set on unstained cells and visualized on a bi-variate histogram (GBM8-EGFP versus CD133-PE). Cells positive for CD133 were quantified using the ratio of cells that fell above the negative cell gates for CD133-PE. A subset of cells was analysed with confocal microscopy to visually confirm the percentage of GFP+ GBM8 cells that stained positive for the CD133-PE.

### Statistical analysis

Data were analysed by Student's *t*-test and paired *t*-test when comparing two groups, and by one-way analysis of variance (ANOVA), two-way ANOVA and repeated measures when comparing more than two groups. Survival times of mouse groups were compared using exact log-rank test. Data were expressed as mean±s.e.m., and differences were considered significant at *P*<0.05.

## Additional information

**How to cite this article:** Bagó, J. R. *et al.* Therapeutically engineered induced neural stem cells are tumour-homing and inhibit progression of glioblastoma. *Nat. Commun.* 7:10593 doi: 10.1038/ncomms10593 (2016).

## Supplementary Material

Supplementary Figures Supplementary Figures 1-7

Supplementary Movie 1 iNSC migration to GBM cells in vitro

Supplementary Movie 2 Single-cell tracing revealing the migratory path

Supplementary Movie 3 Rosetta graph revealing the directed migration of iNSCs

Supplementary Movie 4 iNSC migration to patient-derived GBM8 cells
