# Supplementary material for: Therapeutically engineered induced neural stem cells are tumour-homing and inhibit progression of glioblastoma
Source: Nat Commun. 2016 Feb 2;7:10593. doi: 10.1038/ncomms10593 (PMC4740908; doi:10.1038/ncomms10593)
Supplement: Supplementary Figures — 1-7 [file ncomms10593-s1.pdf]

Supplementary Figures

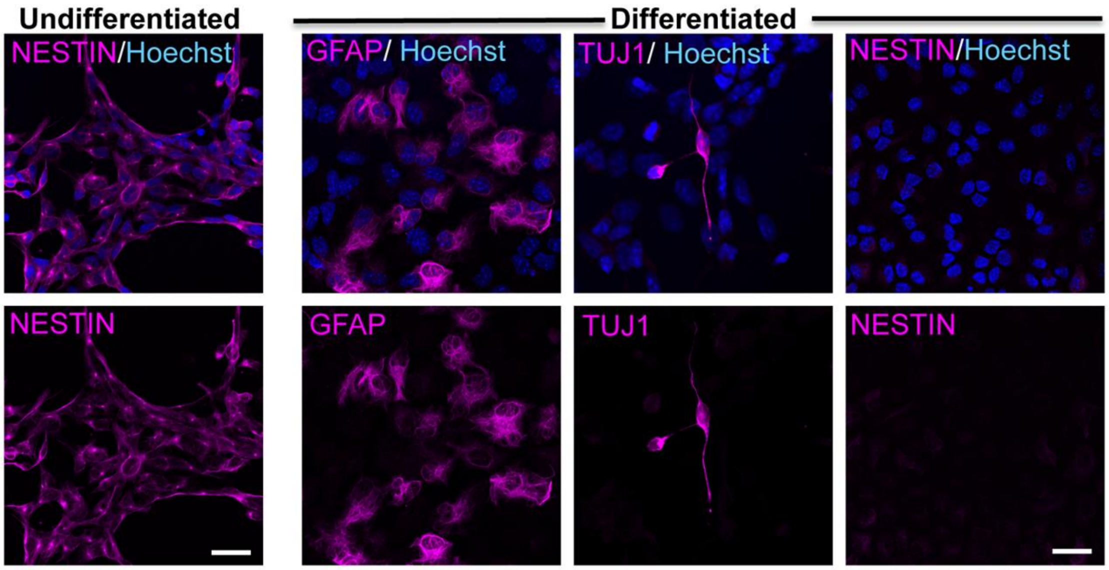

Supplementary Figure 1. Unmodified iNSCs express nestin and differentiate into astrocytes and neurons. Representative images of immunofluorescent staining showing the expression of the NSC marker nestin in iNSCs. Additionally, iNSCs were differentiated by mitogen removal and culturing for 12 days. Staining for GFAP+ astrocytes and Tuj1+ neurons, as well as the levels of nestin, was used to assess differentiation. Fluorescent images showing the red (555 nm) secondary antibody channel alone are shown in the bottom row. Scale bars are 20 μm.

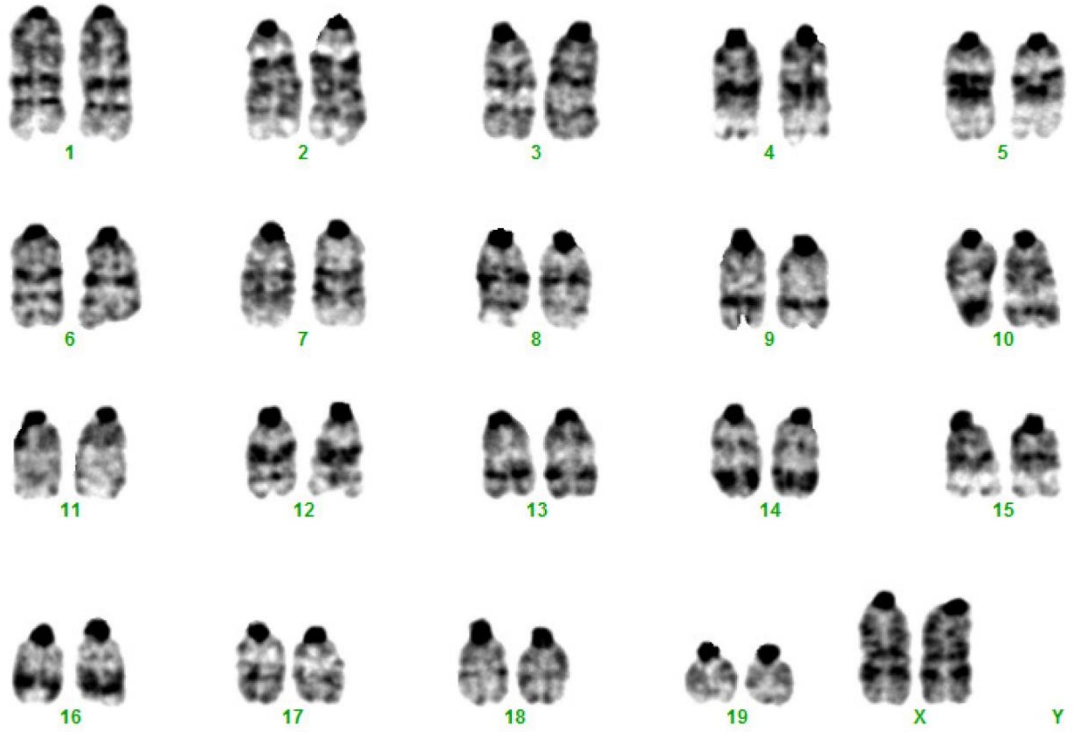

Supplementary Figure 2. Karyotype analysis of engineered iNSCs. Cytogenetic analysis of G-banded metaphase spreads showing the normal karyotype in iNSC-GFPFL.

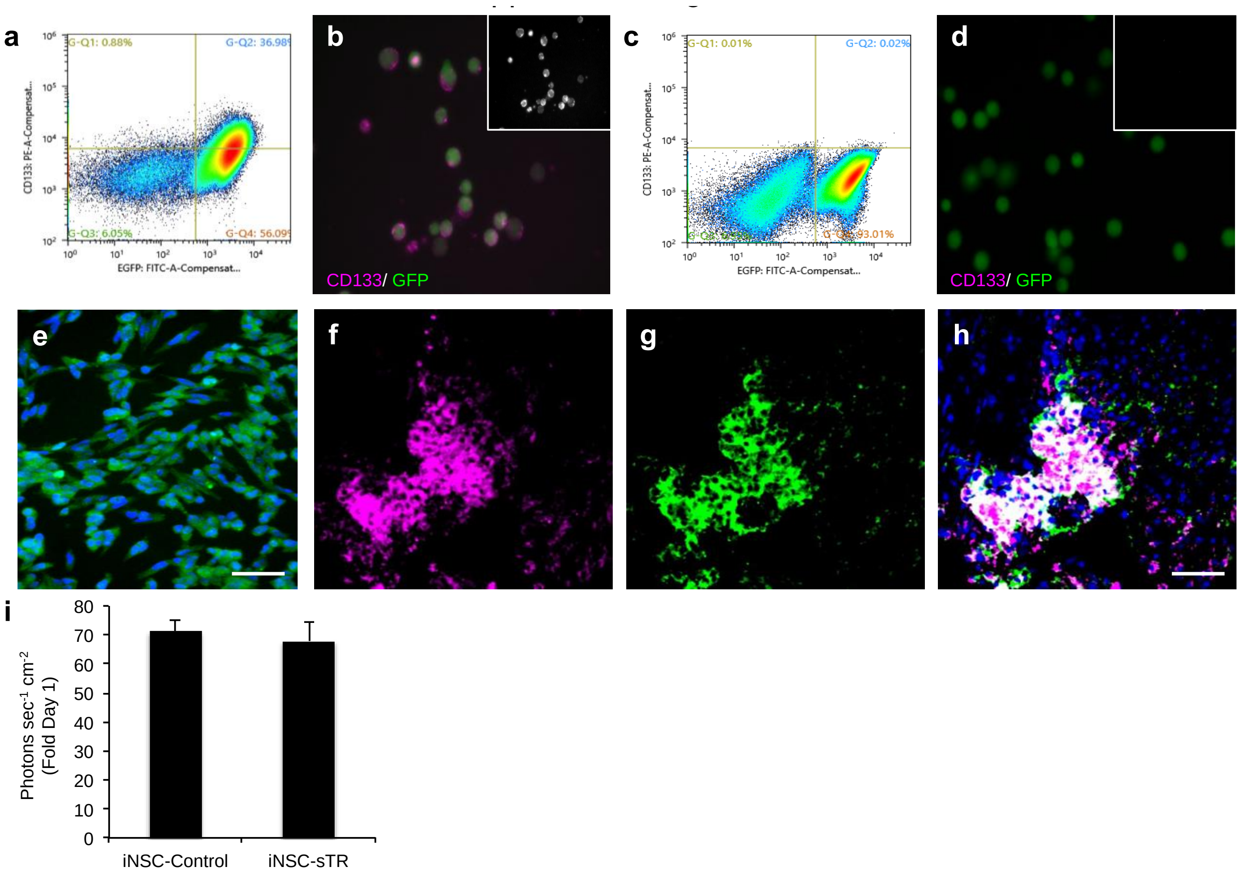

Supplementary Figure 3. Additional characterization of GBM8 cells. (a) Flow cytometric analysis of GFP+ GBM8 cells stained with antibodies against CD133. (b) A portion of CD133-stained GBM8 cells could not be resolved by flow cytometry due to weak signal from the stain. Representative images of the cells at the time of flow analysis show the percentage of GBM8 cells (green) that are positive for CD133 (magenta) determined by fluorescence microscopy analysis. Inset shows the CD133 channel alone. (c-d) Flow cytometry (c) and fluorescent imaging showing CD133 staining in control cells. Inset shows the CD133 channel alone. (e) Representative fluorescent images showing the percentage of GBM8 cells that express CD133 (green) in culture. Hoechst is shown in blue. (f-h) Representative immunofluorescent images of GBM8 xenografts (f) stained with antibodies against CD133 (g). The merged image is shown in panel h. (i) Summary graph quantifying the size of GBM8 tumors at the time of death in iNSC-Control- and iNSC-sTR-treated animals. Scale bars are 30 μm in e and 50 μm in h.

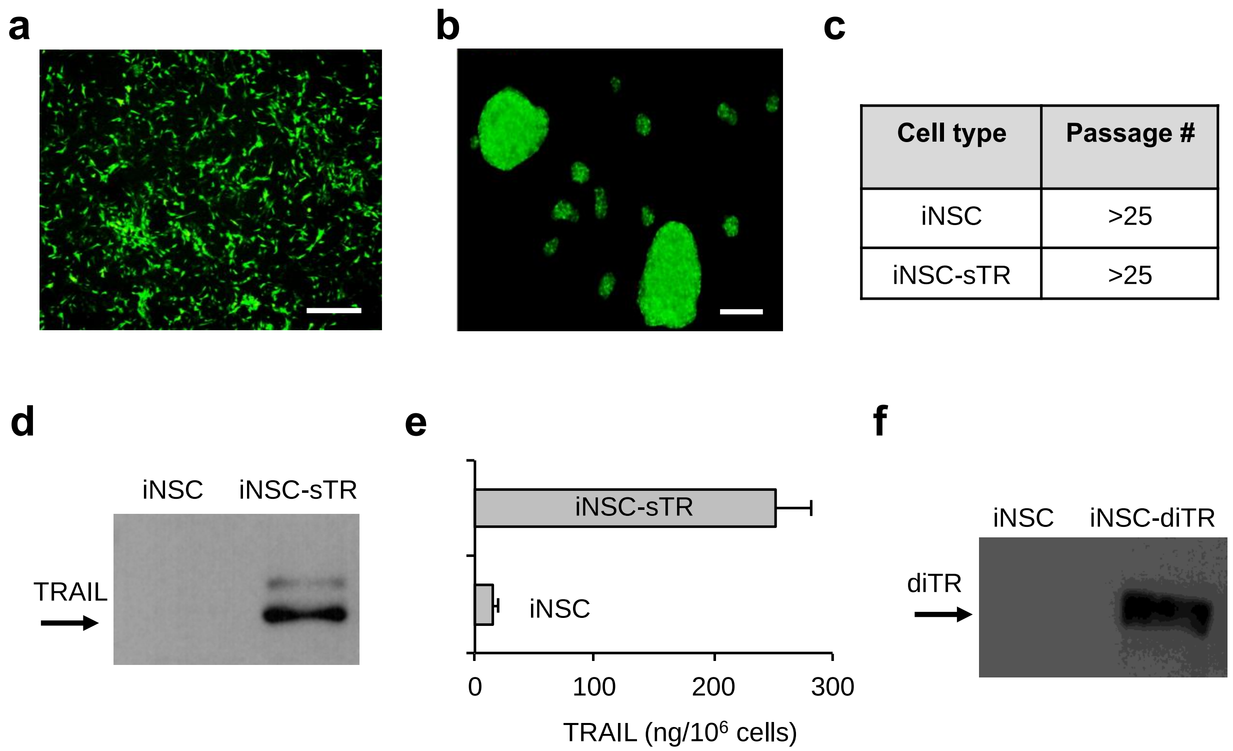

Cell type	Passage #
iNSC	>25
iNSC-sTR	>25

Supplementary Figure 4. Characterization of iNSC-sTR and iNSC-diTR. (a) Representative fluorescent images of iNSC-sTR grown as a mono-layer. (b) Representative fluorescent images showing iNSC-sTR neurospheres. (c) Summary table showing the maximum passage number of iNSC-sTR and unmodified iNSC. (d) Western blot analysis showing the expression of TRAIL protein in iNSC-sTR but not unmodified iNSCs. (e) ELISA assay showing the levels of TRAIL section from iNSC-sTR and control iNSC. (f) Western blot analysis showing the expression of diTR in iNSC-diTR. Scale bars are 20 μm in a and b.

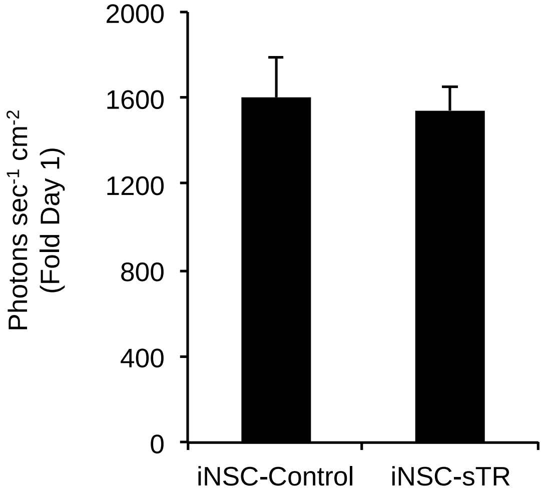

Supplementary Figure 5. U87 tumor regrowth. Summary graph of quantitative bioluminescence imaging that shows the volume of U87 tumors at the time of death in mice treated with iNSC-control or iNSC-sTR.

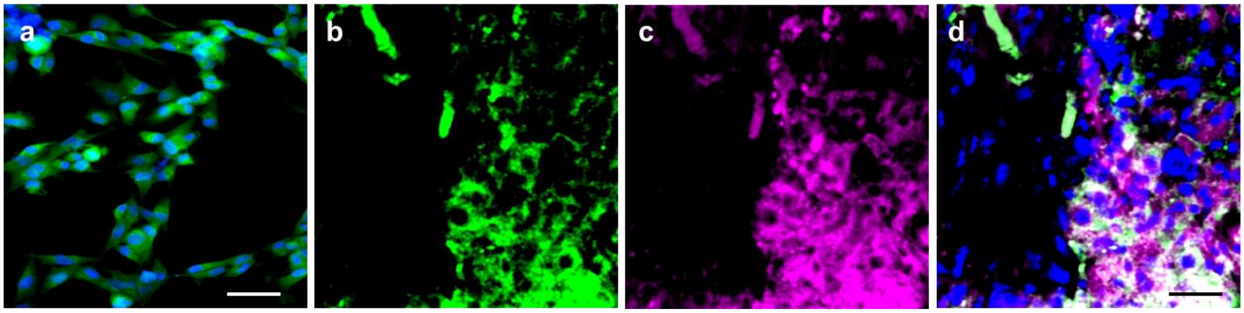

Supplementary Figure 6. CD133 staining of 7063 cells. (a) Representative fluorescent images showing the expression of CD133 (green) by 7063 cells in culture. (b-d) Representative immunofluorescent images of 7063 xenografts (b) stained with antibodies against CD133 (c). The merged image is shown in panel d. Scale bars are 30 μm in a and d.

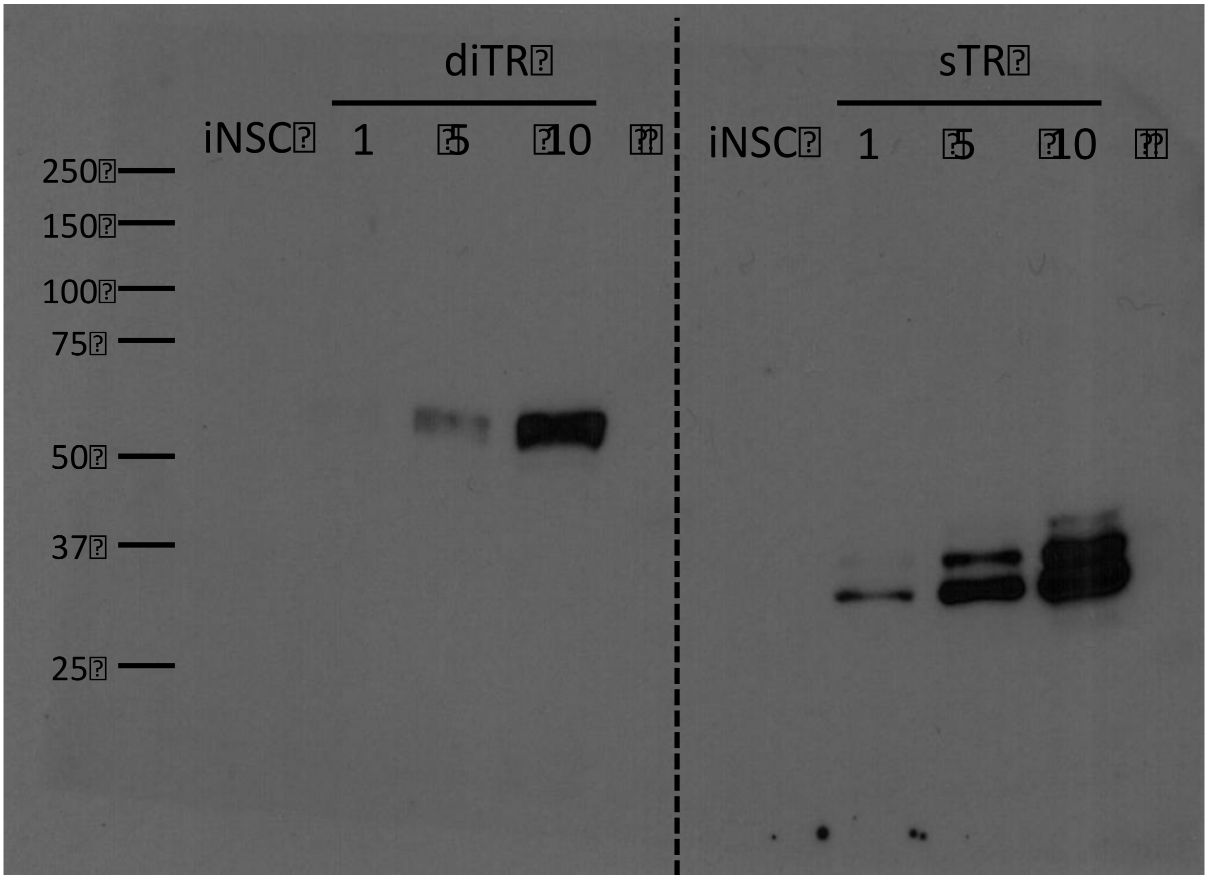

Supplementary Figure 7. sTR and diTR Western blot. Image of the uncropped western blot showing the expression of diTR in or sTR in iNSCs. iNSC=unmodified control. 1, 5, or 10 µg of protein extracts were loaded from iNSC-diTR or iNSC-sTR
